# Polymer Electrolyte/Sulfur Double‐Shelled Anisotropic Reduced Graphene Oxide Lamellar Scaffold Enables Stable and High‐Loading Cathode for Quasi‐Solid‐State Lithium‐Sulfur Batteries

**DOI:** 10.1002/advs.202205424

**Published:** 2022-12-27

**Authors:** Hyun Jung Shin, Sung‐Woo Park, Sangbaek Park, Dong‐Wan Kim

**Affiliations:** ^1^ School of Civil Environmental and Architectural Engineering Korea University Seoul 02841 Republic of Korea; ^2^ Department of Materials Science and Engineering Chungnam National University Daejeon 34134 Republic of Korea

**Keywords:** 3D reduced graphene oxide arrays, free‐standing electrodes, gel‐polymer electrolytes, high areal mass loading, quasi‐solid‐state lithium‐sulfur batteries

## Abstract

Lithium‐sulfur batteries (LSBs) can replace lithium‐ion batteries by delivering a higher specific capacity. However, the areal capacity of current LSBs is low because the intrinsic limitations of sulfur make achieving a high sulfur loading difficult. Herein, the authors report vertically aligned reduced graphene oxide (rGO) with sulfur and poly(ethylene oxide)‐based polymer electrolyte double‐shell layers (VRG@S@PPE) as a high‐loading sulfur cathode. The addition of vapor‐grown carbon fiber (VGCF) into rGO is the key to success, as it allows for gas evacuation from internal nano/micropores without structural collapse, enabling perfect double‐shell layer contact. Owing to the anisotropic rGO lamellar structure that enables straightforward ion/electron transport and provides numerous active sites, sulfur‐infiltrated rGO reinforced via VGCF (VRG@S) exhibits a high capacity of 998 mAh g^−1^ after 100 cycles at 0.1 C under high sulfur loading (6 mg cm^−2^). Interestingly, an additional polymer electrolyte layer further increases the cycle retention (1005 and 718 mAh g^−1^ after 100 cycles at 0.1 and 1 C, respectively), because intimate contact between the solid polymer electrolyte and sulfur could suppress the loss of sulfur due to lithium polysulfide (LPS) shuttling or volume change during lithiation/delithiation. Therefore, it is possible to realize safe and stable quasi‐solid‐state LSBs with high sulfur loading.

## Graphical abstract

Anisotropic reduced‐graphene‐oxide reinforced via vapor‐grown carbon fibers (VRG) become strong enough to uniformly coat their surface with sulfur (S) and poly(ethylene oxide) polymer electrolyte (PPE). Vertically aligned 10 µm‐width micro‐channels provide superior ion/electron transport and accommodate volume expansion during cycling, as well as the thin PPE layer suppresses the shuttling effect, allowing high areal sulfur loading in quasi‐solid‐state lithium‐sulfur batteries.

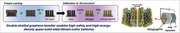

## 1 Introduction

The need for energy storage systems with high energy density has rapidly increased with the increasing popularity of portable electronic devices and the growth of the electric vehicle industry. Given that the energy density of commercial oxide‐based cathodes in lithium‐ion batteries (LIBs) is insufficient for application in large‐scale industries like electric vehicles,^[^ ^1^, ^2^ ^]^ lithium‐sulfur batteries (LSBs) using elemental sulfur as a cathode have been considered a promising alternative because of their high specific capacity (1675 mAh g^−1^) and energy density (≈2600 Wh kg^−1^).^[^ ^3^, ^4^, ^5^, ^6^ ^]^ Moreover, elemental sulfur has the advantage of being extremely abundant and nontoxic. Nevertheless, there are several critical obstacles to the practical industrial application of LSBs as alternatives to LIBs. First, sulfur undergoes excessive volume expansion during the conversion reaction, which results in the collapse of the electrode structure.^[^ ^7^, ^8^ ^]^ Second, because of the low intrinsic electrical conductivity (≈10^−30^ S cm^−1^) of elemental sulfur, a large amount of conductive additives, such as carbon black, are required to obtain ideal performance from LSBs.^[^ ^11^, ^12^ ^]^ Third, when the sulfur cathode reacts with lithium ions during the charge/discharge process, long‐chain polysulfides are produced, which inevitably dissolve in the electrolyte.^[^ ^9^ ^]^ Although dissolved polysulfides impart superior capacity and energy density owing to the transfer of insulating sulfur into the solution phase,^[^ ^10^ ^]^ higher‐order polysulfide intermediates that dissolve in the organic electrolyte are converted into insoluble Li_2_S or Li_2_S_2_ after diffusing to the lithium metal anode, which is called the shuttle effect.^[^ ^11^ ^]^ The shuttle effect leads to the loss of active materials, resulting in low Coulombic efficiency and poor cycling performance of LSBs. These drawbacks of LSBs limit the areal loading mass of the sulfur cathode to less than 1 mg cm^−2^, which is much lower than the commercial standard (20 mg cm^−2^) for LIBs.

Various strategies have been implemented to address the shortcomings of LSBs, such as separator modification,^[^ ^12^, ^13^ ^]^ interlayer insertion,^[^ ^14^, ^15^, ^16^ ^]^ use of a sulfur host material,^[^ ^17^ ^]^ and electrode restructuring.^[^ ^18^, ^19^ ^]^ Among these strategies, modifying the electrode structure to produce a porous 3D structure has various advantages. Owing to its numerous pores, a porous 3D structure can load larger amounts of active materials than a 2D flat structure. Thus, electrode modification to produce a porous 3D structure can be utilized not only for LSBs but also for any other lithium battery systems. Chao et al. fabricated a flexible and free‐standing reduced graphene oxide (rGO) film with a 3D porous structure as a MoS_2_ active material support for LIB anodes.^[^ ^20^ ^]^ In particular, in LSBs, having sufficient space in the structure can suppress the volume expansion of sulfur.^[^ ^21^, ^22^ ^]^ For instance, Hu et al. fabricated a 3D graphene foam nested with an rGO aerogel as a current collector for LSB cathodes.^[^ ^23^ ^]^ The resulting cathode exhibited a high sulfur loading (9.8 mg cm^−2^), sulfur content (83 wt.%), initial capacity (1000 mAh g^−1^), and a capacity of 645 mAh g^−1^ was retained after 350 cycles. However, the capacity rapidly dropped during the initial cycles and decreased by ≈35% during the first 50 cycles. This indicates that improving only the electrode structure is insufficient for suppressing the shuttle effect.

Electrolyte modification is a promising strategy for suppressing the shuttle effect and improving the thermal and electrochemical stability of LSBs.^[^ ^24^ ^]^ Currently, ether‐based liquid organic electrolytes are commonly used as conventional electrolytes for LSBs owing to their high ion conductivity, low reactivity, and good interfacial wettability.^[^ ^25^ ^]^ In contrast to liquid electrolytes, which are flammable and suffer from leakage risks, solid polymer electrolytes (SPEs), which are comprised of polymers like poly(ethylene oxide) (PEO) and poly(vinylidene fluoride) (PVDF), have the advantages of alleviating the shuttle effect and exhibiting superior safety. Despite their advantages, SPEs have some shortcomings as liquid electrolyte substitutes, namely, low ion conductivity at room temperature and poor interfacial contact with the electrode.^[^ ^26^ ^]^ As an alternative, gel polymer electrolytes (GPEs) offer the benefits of both SPEs and liquid electrolytes. GPEs are quasi‐solid‐state electrolytes consisting of high‐molecular‐weight polymer matrices in which liquid electrolytes are dissolved. Therefore, GPEs possess sufficient ion conductivity, safety, stability, and interfacial wettability, providing well‐balanced overall electrolyte performance.^[^ ^27^, ^28^, ^29^ ^]^ Thus, studies on GPEs for LSBs have been actively conducted using various polymer matrices.^[^ ^30^, ^31^, ^32^, ^33^, ^34^ ^]^ However, there are few reports on the simultaneous use of a porous 3D structure electrode and GPE for LSBs. Because sulfur is an insulator, even if a GPE is used for a porous 3D‐structured sulfur cathode, ion conduction will barely occur because the connection between the electrolyte and active materials is poor. Consequently, it is difficult for an active material located at the middle or bottom of a 3D‐structured electrode to participate in a charge/discharge reaction in a GPE system. Therefore, to operate GPEs in porous 3D‐structured electrodes for LSBs, a suitable strategy that can connect the electrolyte to the entire electrode is essential.

**Figure 1:**
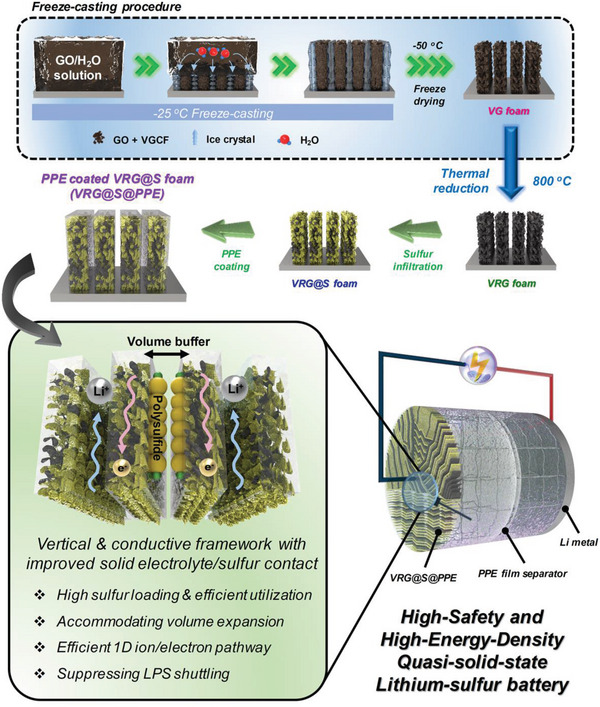
Schematic illustration of the synthetic procedure and detailed internal structure of the VRG@S@PPE cathode.

Herein, we rationally designed a 3D‐structured cathode with a polymer electrolyte inside a quasi‐solid‐state lithium‐sulfur battery (QSSLSB), as shown in **Figure** **1**. First, we fabricated a 3D‐structured free‐standing electrode via the freeze‐casting method using rGO foam as the support and active sulfur as the current collector.^[^ ^35^, ^36^ ^]^ Freeze casting is a facile and cost‐effective method for fabricating porous 3D structures. When a homogeneously casted graphene oxide slurry is placed on a frozen substrate, the ice crystals grow upward from the bottom to the top of the slurry. Subsequently, a vertically aligned 3D structure is created along the growth direction of the ice crystals. In this study, such anisotropic lamellar graphene foam was further supported with vapor‐grown carbon fiber (VGCF), denoted as VRG, to improve mechanical stability and electrical conductivity. As a result, the sulfur was uniformly infiltrated to VRG, denoted as VRG@S, by a facile sonication method without damage to the anisotropic lamellar structure. Subsequently, a PEO polymer electrolyte with dissolved lithium bis(trifluoromethanesulfonyl)imide (LiTFSI) fully infiltrated the entire sulfur‐loaded VRG. The key to success is that the VGCF‐reinforced 3D foam allows the evacuation of gas from the internal pores without structure collapse, allowing for perfect infiltration of the sulfur and carbon slurry and PEO solution, and resulting in the uniform deposition of sulfur and PEO double‐shell layers on the foam surface. This electrode design offers several advantages for QSSLSBs: i) A porous 3D structure can utilize a high sulfur loading and suppress the volume expansion of sulfur, resulting in minimal damage to the electrode structure and leading to higher cycling performance than typical 2D flat electrodes. ii) Vertically aligned channels in the VRG afford short electron/ion transfer pathways, suggesting that they can exhibit high electronic/ionic conductivity. iii) Polymer electrolyte infiltration into the porous 3D structure provides superior contact between the electrode and electrolyte, blocking lithium polysulfide (LPS) shuttling and producing a QSSLSB with outstanding Coulombic efficiency. iv) Because of the high mechanical stability resulting from the infiltration of the sturdy polymer electrolyte into the entire structure, the electrode can be utilized as a free‐standing electrode without an insulating binder material. Accordingly, we realized superior QSSLSB performance and demonstrated that this simple strategy can be applied to other energy‐related materials.

## 2 Results and Discussions

**Figure 2:**
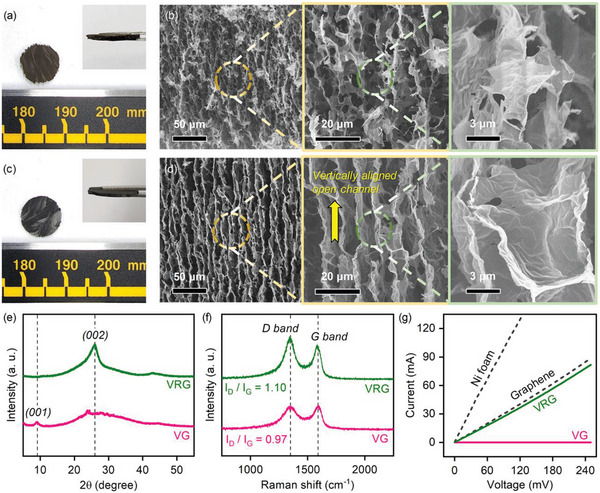
Photographs of the top‐ and side‐view (inset) of the a) VG and c) VRG foams. High to low magnification SEM images of the b) VG and d) VRG foams. e) XRD patterns, f) Raman spectra, and g) *I–V* curves of the VG and VRG foams.

**Figure** **2**a shows photographs of the top and side views of the VGCF/GO (VG) foam fabricated by the freeze‐casting process. The VG foam had a rough surface and a dark brown color with a thickness of 500 µm (inset of Figure 2a). Figure 2b shows cross‐sectional scanning electron microscopy (SEM) images of the VG foam. The VG foam consisted of graphene sheets with a width of approximately 10–15 µm arranged in parallel. Continuous and vertical empty spaces were formed between the graphene sheets owing to this parallel arrangement, which provided a vertically aligned anisotropic lamellar scaffold. GO was transformed into rGO by the heat treatment of VG foam at 800 °C in an Ar/H_2_ atmosphere, creating a VGCF/rGO (VRG) foam as a free‐standing conductive 3D framework. Although the surface color of the VRG foam changed to dark grey, the surface patterns and thickness of the foam were maintained, as shown in Figure 2c. The internal 3D structure also did not collapse during thermal reduction; instead, the vertically aligned open channel was more robust and clearly conserved by annealing (Figure 2d). Such vertically aligned open channels with a diameter of approximately 10–20 µm can provide several benefits to LSBs as unique cathode scaffolds, such as 1) a straightforward ion/electron pathway through vertically aligned graphene facilitating rapid electron/ion transfer/transport, 2) a wide surface area for high sulfur loading, and 3) accommodation for the volume expansion of sulfur. Owing to its ultralight nature (≈1 mg), the density of VRG was only 0.039 g cm^−3^, indicating that the VRG interior was mostly empty.

Figure 2e shows the X‐ray diffraction (XRD) patterns of the VG and VRG foams. The peak appearing around 10° in the XRD pattern of the VG foam corresponds to a (001) plane carbon peak with a d‐spacing of 8 Å, indicating the presence of typical graphene oxide.^[^ ^37^, ^38^ ^]^ In the XRD pattern of the thermally reduced VRG foam, the (001) plane carbon peak disappeared, and a new peak was observed around 25°. This new peak indicates the formation of a (002) plane with a d‐spacing of 4 Å, suggesting that the interplanar spacing of the graphene crystal plane was reduced due to the elimination of oxygen‐containing groups during the thermal reduction process. The presence of rGO was also confirmed by Raman spectroscopy (Figure 2f). The peaks around 1350 and 1600 cm^−1^ in the Raman spectra are attributable to the D and G peaks, respectively. The D peak indicates the crystal defect/disorder state of graphene oxide, and the G peak denotes sp^2^ bonding between carbon atoms.^[^ ^39^ ^]^ The degree of reduction of graphene oxide was evaluated by comparing the intensity ratio of the D and G peaks (*I*_D_/*I*_G_) of each sample. The *I*_D_/*I*_G_ ratios of the VG and VRG foams were calculated to be 0.97 and 1.10, respectively, suggesting that defects occurred in the crystal plane due to the elimination of oxygen groups in the crystalline structure, and the number of sp^2^ bonds between the carbon atoms increased. Therefore, it was demonstrated that the graphene oxide in the VG foam was successfully transformed to rGO through thermal reduction. The electrical conductivity of the electrode is an essential factor for its application as a free‐standing current collector for LSBs. Figure 2g shows the *I–V* curves for each sample with the same size. The VG foam appeared to be an insulator with little current flow even at a relatively high voltage. In contrast, the VRG foam exhibited a relatively high current flow similar to that of commercial graphene, although it was lower than that of the metal Ni foam. This result demonstrates that the VRG foam possesses sufficient electrical conductivity for use as an electrode in LSBs.

**Figure 3:**
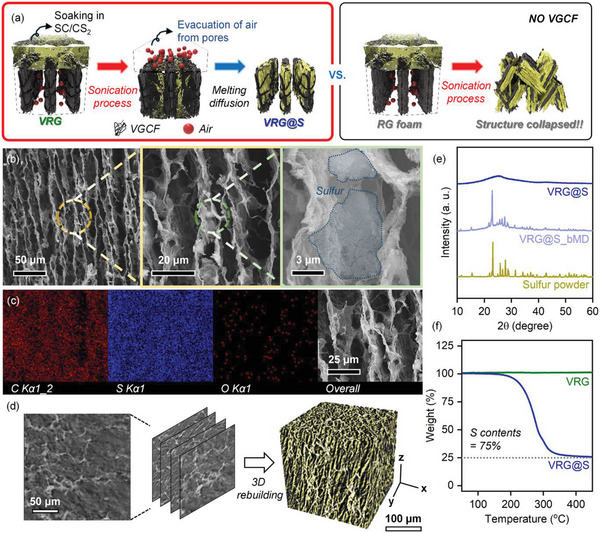
a) Schematic illustration of the principle of sulfur infiltration into the VRG foam through sonication. b) High to low magnification SEM images and c) SEM‐EDS mapping of the VRG@S foam. d) XRM 2D image and reconstructed 3D projection of the VRG@S foam. e) XRD patterns and f) TGA curves of the VRG@S foam.

A sulfur shell layer was deposited on the surface of the VRG foam to create a uniform VRG@S core‐shell structure. Among the various methods, sonication was the most suitable for uniformly distributing the active sulfur material inside the VRG foam and eliminating air in the micropores of the VRG foam (Data S1 and Figures S1 and S2, Supporting Information). **Figure** **3**a shows a schematic illustration of the synthesis of VRG@S foam through sonication and melting diffusion. Sonication was performed by immersing the VRG foam in S‐C/CS_2_ and sonicating for approximately 5 min, and the process was repeated thrice. During sonication, air bubbles were vigorously evacuated from the microscopic pores of the VRG foam, and the active materials were homogeneously dispersed in the air bubble vacancies. The key to success is to enhance the mechanical strength of the rGO foam by incorporating 1D VGCF in a GO suspension at the foam synthesis stage. Without VGCF, the rGO foam collapsed easily during sonication. In fact, rGO foams with and without VGCF (i.e., VRG and RG foams, respectively) were immersed in the S‐C/CS_2_ solution and sonicated for 5 min. The VRG foam maintained its structure without any destruction, whereas the RG foam structure collapsed (Videos S1 and S2, Supporting Information). The disintegration of the RG foam was initiated only 1 min after sonication and the foam was almost destroyed after 3 min, whereas the VRG foam supported by VGCF did not collapse during the entire sonication process and maintained its original state (Data S2 and Figures S3 and S4, Supporting Information). It clearly reveals the enhanced mechanical robustness of VRG foam via mechanically mixed VGCF and rGO (Data S3 and Figure S5, Supporting Information). Consequently, the VGCF‐reinforced 3D foam allows for the evacuation of gas from the internal pores without collapse of the structure during sonication, resulting in perfect infiltration of S‐C/CS_2_ and uniform deposition of sulfur and carbon agents on the foam. The VRG foam impregnated with sulfur was synthesized into VRG@S foam through melting diffusion at 155 °C for 12 h. The melting diffusion process resulted in a more uniform distribution of the sulfur shell layer in the foam and improved contact with rGO.

Figure 3b shows SEM images of the sulfur‐infiltrated VRG foam (denoted as VRG@S foam). As shown in the high‐magnification image in Figure 3b, the sulfur particles were homogeneously coated on the rGO sheet. The vertically aligned structure of the foam was maintained without any destruction even after sulfur infiltration. In addition, the energy‐dispersive X‐ray spectroscopy (EDS) elemental mapping confirmed the uniform distribution of sulfur throughout the rGO sheet in the VRG@S foam (Figure 3c). Oxygen was barely detected owing to the previous thermal reduction process. Moreover, the X‐ray microtomography (XRM) image of the VRG@S foam also showed that the sulfur particles (white parts) were firmly anchored to the rGO sheet, as shown in the cross‐sectional XRM 2D image (Figure 3d). Furthermore, an XRM 3D image was reconstructed by stacking thousands of 2D images to investigate the detailed structure of the VRG@S foam. Similarly to the 2D images, homogeneous sulfur distribution was confirmed by the XRM 3D images as well, wherein the rGO sheet is shown in dark gray and sulfur in yellow. The yellow sulfur part is compactly distributed on the gray rGO sheet part, as shown in the XRM 3D image. Therefore, it is clear that sulfur was distributed homogeneously along the vertically aligned rGO sheets due to sonication and melting diffusion.

Figure 3e shows XRD patterns of the sulfur powder and VRG@S foam before and after the melting diffusion process (denoted as VRG@S_bMD and VRG@S, respectively). Crystalline sulfur peaks were still observed in the XRD pattern of VRG@S_bMD, whereas they completely disappeared and only rGO peaks remained after melting diffusion. This means that the active sulfur penetrated intensely inside the foam, and residual sulfur on the surface, which inhibits the electrical conductivity of the cathode, was successfully removed during the melting diffusion process. According to nitrogen adsorption‐desorption isotherm results, the BET surface area of VRG foam was decreased from 233.6 to 219.6 m^2^ g^−1^ after sulfur loading (Figure S6a,b, Supporting Information). Furthermore, as shown in pore size distribution, the proportion of pores with smaller diameters was increased after sulfur infiltration to VRG (Figure S6c,d, Supporting Information). Thus, the smaller surface area of VRG@S than VRG is due to the decreased pore size after sulfur loading, because the infiltrated sulfur accumulates in the rGO sheet and blocks the pores. The thermal decomposition properties of the VRG and VRG@S foams were investigated using thermogravimetric analysis (TGA). The VRG foam exhibited no weight loss throughout the temperature range, whereas the VRG@S foam exhibited a single weight loss in the temperature range of approximately 200–300 °C (Figure 3f). Because the weight loss was attributed to the evaporation of sulfur from the VRG@S foam, the sulfur content of the VRG@S foam was calculated to be ≈75% based on the TGA results.

**Figure 4:**
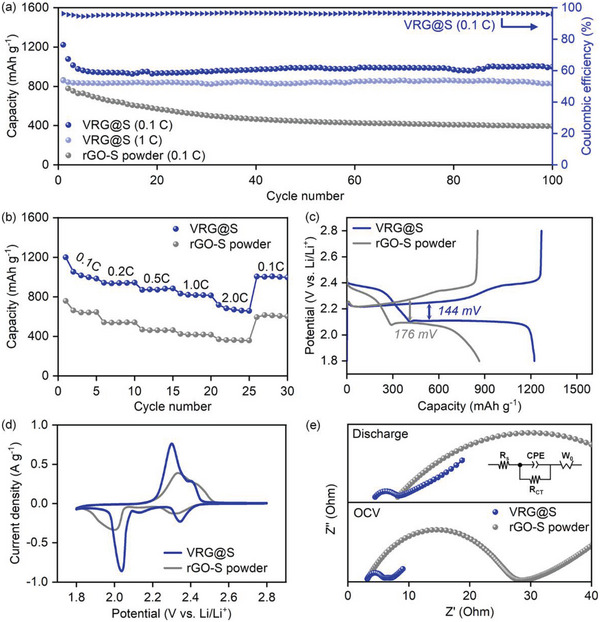
Electrochemical properties of VRG@S. a) Long‐term stability at 0.1 and 1.0 C for LSB cells, b) cycling plot under C‐rates of 0.1–2.0, c) GCD curves of the first cycle at 0.1 C, d) CV profiles, and e) Nyquist plot of the fresh and discharged states using VRG@S foam and rGO‐S powder cathodes.

The electrochemical performance of the VRG@S foam as a cathode in LSBs was investigated (**Figure** **4**). The rGO‐S composite powder, fabricated by grinding VRG@S foam, was also tested as a control sample. The loading mass of the active material was approximately 6 mg cm^−2^ for both the VRG@S foam and rGO‐S powder, which is higher than that of typical sulfur electrodes (1–2 mg cm^−2^). Figure 4a and b show the long‐term cycle performance at a rate of 0.1 C and the rate capability, respectively, for the VRG@S foam and rGO‐S powder cathodes. As shown in Figure 4a, the specific discharge capacity during the first cycle of the VRG@S foam was quite high at 1222 mAh g^−1^, while the rGO‐S powder exhibited a relatively low capacity of 863 mAh g^−1^. This performance difference was also observed in cycle retention. The VRG@S foam retained a constant capacity of approximately 1000 mAh g^−1^ throughout the cycling process, except for a slight capacity fading during the initial cycles. In contrast, the capacity of the rGO‐S powder decreased steadily and dropped to 400 mAh g^−1^ after the 100th cycle, suggesting that the LPS shuttling effect was more severe with the rGO‐S powder. This reveals that a VRG@S foam with a 3D structure and high pore volume enables most of the active materials to easily participate in the electrochemical reaction. Post‐mortem SEM and EDS analysis present that such a unique structure of VRG@S electrode was not damaged after cycling (Figure S7, Supporting Information). Furthermore, the VRG@S foam showed a higher rate performance than the VRG@S powder at all rates from 0.1 to 2.0 C (Figure 4b). In particular, a high specific discharge capacity of approximately 800 mAh g^−1^ was obtained at 1 C, which remained unchanged for 100 cycles (Figure 4a). The capacity, C‐rate, and cycle retention of VRG@S are much higher than those of previously reported 3D porous structures in LSBs, which show one of the highest levels among the previously reported high‐loading sulfur electrodes for LSBs (Table S1, Supporting Information).

Further electrochemical analysis was conducted to understand the effect of the anisotropic rGO lamellar structure on LSBs. Figure 4c presents the galvanostatic charge/discharge (GCD) curves at 0.1 C. The voltage polarizations of the VRG@S foam and rGO‐S powder were 144 and 176 mV, respectively, in the first cycle. The VRG@S foam exhibited lower voltage polarization than the rGO‐S powder, suggesting that the VRG@S foam cathode provides efficient electrochemical reaction sites and electron/ion pathways through its unique vertically aligned lamellar structure. Figure 4d shows the cyclic voltammetry (CV) profiles of the VRG@S foam and rGO‐S powder at a rate of 0.05 mV s^−1^. Both the VRG@S foam and rGO‐S powder electrodes showed two anodic oxidation peaks and two cathodic reduction peaks, which corresponded to the voltage range of the plateau that emerged in the GCD curves. In addition, the small peak at 2.1 V is observed in VRG@S, which is commonly observed in the sulfur cathode with high contents of carbon‐based material. It relates to unstable intermediate polysulfide species formed during the reduction process at ≈2.4 V.^[^ ^40^ ^]^ The anodic oxidation peak of VRG@S foam appeared at 2.30/2.41 V, and the cathodic reduction peak appeared at 2.04/2.34 V. The voltage gap between the anodic oxidation peak and cathodic reduction peak of the VRG@S foam was lower than that between the corresponding peaks of the rGO‐S powder. This suggests that the LPS conversion reaction in the VRG@S foam cathode was more reversible than that in the rGO‐S powder cathode. Electrochemical impedance spectroscopy (EIS) was also performed. Figure 4e shows Nyquist plots for the fresh and discharged states of the VRG@S foam and rGO‐S powder cathodes. The appearance of a semicircle in the high‐frequency region in the Nyquist plot indicates charge transfer resistance (*R*_ct_), which was much smaller for the VRG@S foam than for the rGO‐S powder in both the fresh and discharged states (Table S2, Supporting Information). This shows that charge transfer for LPS conversion in the VRG@S foam is superior to that in the rGO‐S powder cathode. The above results show that the anisotropic rGO lamellar scaffold is beneficial for the utilization of sulfur in high‐loading cathodes.

**Figure 5:**
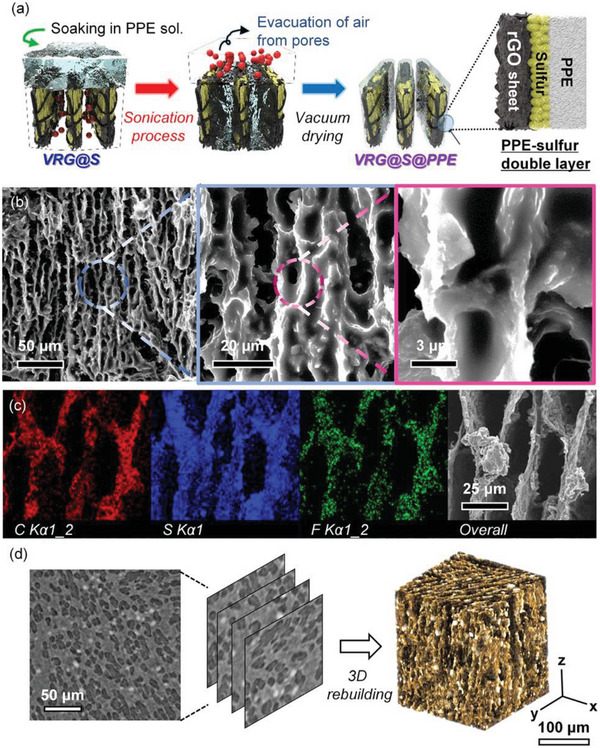
a) Schematic illustration of the principle of PPE infiltration into VRG@S through sonication and the structure of VRG@S@PPE. b) High to low magnification SEM images and c) SEM‐EDS mapping of VRG@S@PPE. d) XRM 2D image and reconstructed 3D projection of VRG@S@PPE.

The drawback of VRG@S foam as a cathode in LSBs is the initial capacity loss (Figure 4a,b), which may be attributed to the dissolution of LPS into the electrolyte. To address this, a PEO‐based polymer electrolyte (PPE) was coated onto the surface of the VRG@S foam (**Figure** **5**a). Similar to the sulfur infiltration method, sonication was the best method to eliminate air bubbles from the microscopic pores of the VRG@S foam and to infiltrate the PPE solution into the VRG@S foam (Data S4 and Figures S8—S10, Supporting Information). As a result, the VRG foam had double‐shell layers composed of sulfur and PPE (denoted as VRG@S@PPE), which can improve electrode‐electrolyte contact and suppress LPS shuttling. Figure 5b,c show SEM and EDS images of the VRG@S@PPE material. The internal structure of VRG@S@PPE was confirmed by SEM. As shown in the high‐magnification SEM image (Figure 5b), the sulfur‐anchored rGO sheet was compactly wrapped by PPE. Despite the injection of heavy and high‐viscosity PPE through a harsh sonication procedure, the rGO sheets maintained their vertically aligned structure without collapse. Additionally, the uniform infiltration of PPE was investigated using EDS elemental mapping (Figure 5c). Fluorine, which is a component of PPE, was detected throughout the VRG@S@PPE structure. Sulfur was also evenly distributed without loss during PPE infiltration. Moreover, as shown in the cross‐sectional XRM 2D image of VRG@S@PPE (Figure 5d), the PPE (gray region) was evenly distributed and covered the rGO sheet and sulfur (dark gray region). Furthermore, the uniform distribution of PPE was confirmed more clearly via the reconstructed XRM 3D image. The orange region, indicating PPE, was densely distributed throughout the interior of VRG@S. In addition, the sonication method even allowed the high concentration of PPE to be infiltrated in VRG foam, whereas most PPE just accumulated on the surface of VRG foam by the soaking/vacuum infiltration method (Figure S10, Supporting Information). These results suggest that PPE infiltration by sonication can evacuate the gas from the internal pores, resulting in perfect infiltration of the polymer electrolyte and uniform deposition of the polymer electrolyte on the foam.

Notably, VRG@S@PPE showed much better initial capacity retention (Figure S11, Supporting Information) than the bare VRG@S foam or VRG@S foam with PPE prepared by only soaking and vacuum drying (Figure S9, Supporting Information). Interestingly, high capacity retention was obtained with a very thin PPE coating layer through the addition of 3 mg of PPE (Figure S11b, Supporting Information). The capacity retention of VRG@S@PPE decreased as the amount of infiltrated PPE increased (Figure S11c—e, Supporting Information). As shown in the discharge curves, a thicker PPE layer lowers the discharge plateau, implying an increase in the resistance of the low‐ionic‐conductivity polymer electrolyte layer. This result indicates that the sonication method, enabled by the enhancement in the mechanical strength of rGO foam brought about by VGCF, is beneficial for creating a thin and uniform PPE shell layer without leading to the concentration of PPE in specific regions or the remaining pores.

**Figure 6:**
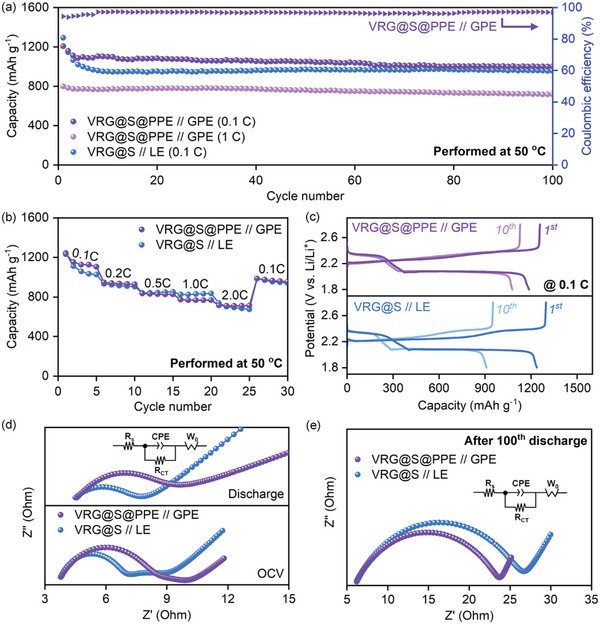
Electrochemical properties of VRG@S@PPE for QSSLSB cells. a) Long‐term stability at 0.1 and 1.0 C, b) cycling plot under C‐rates of 0.1–2.0, c) GCD curves of the first and 10th cycles at 0.1 C. Nyquist plots at the open‐circuit voltage (OCV) and discharged stage in the d) fresh state and e) after 100 cycles.

The electrochemical performance of VRG@S@PPE with 3 mg PPE was further investigated in a QSSLSB cell using a PPE film (150 µm thickness) as a solid polymer electrolyte separator (denoted as VRG@S@PPE//GPE). The cell using the VRG@S foam without a PPE shell layer and PPE separator was also evaluated as a control sample (denoted as VRG@S//LE). **Figure** **6**a,b show the long‐term cycle performance at 0.1 C and the corresponding GCD curves of VRG@S@PPE//GPE, respectively. As shown in Figure 6a, VRG@S@PPE//GPE and VRG@S//LE exhibited similar initial discharge capacities of 1207 and 1292 mAh g^−1^ at 0.1 C, respectively, although they showed differences in cycle retention. During the first 10 cycles, VRG@S@PPE//GPE maintained a high discharge capacity of 1080 mAh g^−1^ with a capacity retention of 91%, whereas the discharge capacity of VRG@S//LE rapidly dropped to 955 mAh g^−1^ with a capacity retention of 74%. Accordingly, VRG@S@PPE//GPE exhibited a low capacity fading rate (0.17% per cycle) over 100 cycles, whereas VRG@S//LE exhibited a high capacity fading rate (0.26% per cycle). As a result, the VRG@S@PPE//GPE exhibits a much lower polarization as well as higher capacity than VRG@S//LE after 100 cycles (Figure S12, Supporting Information). This is attributed that the diffusion of polysulfide toward the lithium anode (LPS shuttling) was effectively suppressed by the PPE shell layer and PPE separator. It is well‐known that polymer electrolytes have the ability to suppress LPS shuttling due to the repulsive electrostatic interactions between polysulfide anions and their negatively charged polymer matrix.^[^ ^41^ ^]^ As direct evidence, the LPS blocking test was conducted using an H‐type glass cell (Figure S13, Supporting Information), revealing that LPSs diffused through the VRG foam, whereas LPSs hardly diffused through PPE‐coated VRG foam. Moreover, VRG@S@PPE//GPE exhibited discharge capacities of 1104, 927, 825, 766, and 712 mAh g^−1^ at 0.1, 0.2, 0.5, 1.0, and 2.0 C respectively, which were similar to those of VRG@S//LE (Figure 6b). This means that VRG@S@PPE//GPE retained the high rate capability of VRG@S despite the low‐ionic‐conductivity polymer layer. This was attributed to the intimate contact of the thin PPE shell layer with VRG@S, which minimized the increase in resistance caused by the polymer electrolyte layer. EIS analysis was conducted before and after cycling at 50 °C. Figure 6d shows Nyquist plots of VRG@S@PPE//GPE and VRG@S//LE in the fresh state and after the first discharged state. The semicircle indicating *R*_ct_ was slightly smaller for VRG@S//LE in both the fresh and discharge states. However, as shown in Figure 6e, the semicircle of VRG@S//LE became very large, but the semicircle of VRG@S@PPE//GPE slightly changed after 100 cycles (Table S2, Supporting Information). It suggests that VRG@S@PPE//GPE exhibited an efficient electrochemical reaction owing to the LPS diffusion‐blocking effect. The slight change of *R*_ct_ in VRG@S@PPE//GPE was still observed because the volume expansion/shrinkage of sulfur cannot be completely suppressed via a thin PPE coating layer. Remarkably, VRG@S@PPE//GPE achieved long‐term cycle stability with 1005 mAh g^−1^ at 0.1 C after 100 cycles, which is superior to that of previously reported polymer‐electrolyte‐based LSBs with high sulfur loading (Table S3, Supporting Information). Therefore, these results suggest that VRG@S@PPE affords high capacity, C‐rate, and cycle retention even under high sulfur loading (6 mg cm^−2^) and high operating temperatures (50 °C), demonstrating a stable and high‐energy‐density QSSLSB.

## 3 Conclusion

In summary, we demonstrated an anisotropic rGO lamellar structure wrapped with sulfur and polymer electrolyte double‐shell layers as a stable and high‐loading sulfur cathode for QSSLSBs. The free‐standing, conductive, and vertically aligned VRG lamellar framework was fabricated using a freeze‐casting and ice‐templating method. In addition, sulfur and PPE double‐shell layers were successively formed on the VRG scaffold via a simple sonication method. The key to achieving intimate contact with the double‐shell layers is the incorporation of 1D VGCF, which enhances the mechanical strength of the rGO foam. This allows for the infiltration of sulfur and PPE solutions while removing trapped air inside nano/micropores without leading to the collapse of the complex structure during sonication. As a result, VRG@S exhibited high capacities of 998 and 825 mAh g^−1^ after 100 cycles at 0.1 and 1.0 C, respectively, even under a high sulfur loading (6 mg cm^−2^) for LSBs. This is attributed to the unique anisotropic lamellar structure, which provides straightforward Li‐ion channels/electron pathways as well as 3D light and porous scaffolds. Interestingly, the cycle stability was improved by the second PPE shell layer because intimate contact between the solid polymer electrolyte and sulfur could suppress the loss of sulfur active material due to dissolution and diffusion of LPS or volume expansion of sulfur during cycling. Consequently, VRG@S@PPE achieved a superior capacity of 1005 mAh g^−1^ after 100 cycles at 0.1 C and a high capacity retention of 83.3%. Furthermore, it showed remarkable long‐term cycle stability of 718 mAh g^−1^ after 100 cycles at 1 C, which is one of the highest levels among previously reported high‐loading and QSSLSBs. These findings point toward the strong synergy of the combination of multi‐shell layers and vertically aligned open channels, which will lead to new avenues for high active material loading of LSBs, safe quasi‐solid‐state batteries, other energy storage/conversion devices, and electrochemical catalysis applications.

## 4 Experimental Section

### Preparation of VRG 3D Foam

First, 10 mg of VGCF (Showa Denko K. K., Japan), 120 mg of F127 (bioreagent, Sigma‐Aldrich), and 60 µL of ammonia solution (25–30%, Samchun) were dispersed in 20 mL of deionized water using an ultrasonic processor for 10 min. Then, 80 mg of urea (99.3+ %, Alfa Aesar), as a nitrogen‐doping source, was dispersed in a VGCF suspension by stirring for 1 h. Subsequently, 10 mL of the VGCF suspension and 6 g of graphene oxide gel (2 wt%, N002‐PS, Angstron Materials) were homogeneously mixed by stirring for 2 h to obtain a VG suspension. The as‐prepared VG suspension was then cast on an alumina plate (*d* = 1 mm) to produce a 500‐µm‐thick layer using a doctor's blade. The casted VG suspension was transferred to a SUS plate, which was cooled to −25 °C using liquid nitrogen to achieve rapid freezing. The frozen VG was dried by lyophilization for 24 h. The obtained foam was peeled off the alumina plate and cut into a square of size 15 × 15 mm. Subsequently, 10 pieces of VG foam were placed in an alumina boat and placed in the center of a quartz tube. Thermal reduction of the VG foam was carried out at 800 °C for 2 h, and subsequently, the carbon coating process was performed for 1 h at the same temperature using acetone as the carbon source, to improve the electrical conductivity of the electrode.^[^ ^35^, ^36^ ^]^ All the thermal processes were carried out under an Ar/H_2_ (95/5 sccm) flow. The obtained samples were punched into a disk with a diameter of 10 mm and denoted as VRG foam (VGCF/rGO foam).

### Infiltration of Sulfur and PPE in the VRG Foam

First, 2.5 g of elemental sulfur and 0.1 g and Ketjen black were mixed and ground using a mortar and pestle. The mixed powder was dispersed in 30 mL of carbon disulfide (CS_2_, ≥ 99.9%, Sigma‐Aldrich) solvent and sonicated for 2 h. The as‐obtained suspension was denoted as S‐C/CS_2_. The VRG foam was immersed in the S‐C/CS_2_ suspension, sonicated for 5 min, and dried for 5 min. The sonication and drying processes were repeated thrice. The as‐obtained sulfur‐infiltrated VRG foam (denoted as VRG@S_bMD) was completely dried residual CS_2_ solvent in a vacuum oven for 2 h at room temperature. The VRG@S_bMD material was placed in a 200 mL Teflon liner and the inside atmosphere was replaced with Ar gas. Subsequently, the melting diffusion process of VRG@S_bMD was conducted at 155 °C for 12 h. The obtained samples were stored in an Ar‐filled glove box and denoted as VRG@S foam. PEO (*M*_w_ = 100 000, Sigma‐Aldrich) and LiTFSI (Alfa Aesar) were completely dried at 50 and 110 °C, respectively, in a vacuum oven for 24 h prior to use. PEO and LiTFSI were dissolved in acetonitrile solvent at an EO/Li ratio of 10 and sonicated for 3 h in a completely sealed state to block air. The concentration of the PPE solution was controlled by varying the amount of the acetonitrile solvent. The VRG@S foam was immersed in PPE solution and sonicated for 3 min. The sample was completely dried in a vacuum oven for 24 h to fully evaporate the acetonitrile. The drying process was conducted at room temperature to prevent the sublimation of sulfur in the VRG@S foam, and the obtained sample was denoted as VRG@S@PPE.

### Fabrication of the PPE Film

PEO (*M*_w_ = 600 000, Sigma‐Aldrich) and LiTFSI were completely dried at 50 and 110 °C, respectively, in a vacuum oven for 24 h prior to use. PEO and LiTFSI were dissolved in acetonitrile at an EO/Li ratio of 10 and vigorously stirred for 24 h in an Ar‐filled glove box to obtain a homogenous PEO solution. Celgard 2400 separators were placed in a Teflon dish, and the PEO solution was subsequently poured over them. Then, the PEO solution in the Teflon dish was placed in a vacuum oven and completely evaporated at 50 °C for 48 h. The as‐obtained PPE Celgard was punched into a disk with a diameter of 18 mm and denoted as PPE film.

### Material Characterization

The microstructure, morphological features, and elemental mapping were analyzed using field‐emission scanning electron microscopy (FE‐SEM; SU‐70, Hitachi, Japan) equipped with an EDS system. The crystal structures were analyzed by XRD (MiniFlex 600, Rigaku, Japan). The graphene structures and compositions were evaluated using Raman spectroscopy (LabRam ARAMIS IR2, HORIBA JOBIN YVON, Japan). The 3D structures were investigated using XRM (Xradia 620 Versa, Carl Zeiss, USA). The carbon and sulfur contents were calculated using TGA (STA 449F3, NETZSCH, Germany).

### Electrochemical Characterization

The electrochemical performances for LSBs were evaluated by assembling a CR2032‐type coin cell, which consisted of the as‐prepared VRG‐based cathode, a Celgard 2400 separator, a Li metal anode, and a liquid electrolyte (1 m LiTFSI and 0.2 m LiNO_3_ dissolved in a 1:1 volumetric mixture of DOL/DME). The coin cell for the QSSLSB consisted of the as‐prepared VRG@S@PPE cathode, a PPE film as a separator, a Li metal anode, and a liquid electrolyte. The 60 µL of liquid electrolyte was directly dropped on the VRG@S@PPE, and the ratio of electrolyte to sulfur was 12.8 µL mgS^−1^. All the coin cells were assembled in an Ar‐filled glove box. The rGO‐S powder cathode was fabricated from a slurry that consisted of 80% ground VRG@S foam, 10% carbon black (Ketjen Black EC‐600JD), and 10% PVDF (Sigma‐Aldrich). The slurry was cast on a 10‐mm‐diameter Al foil disk. GCD and CV tests were performed using an assembled cell in the voltage range of 1.8–2.8 V (vs Li/Li^+^) at various current rates (1 C = 1675 mAh g^−1^). Cell tests were performed using an automatic battery cycler (WonATech, Korea). EIS was performed in the frequency range of 10^−2^–10^5^ Hz with an amplitude of 5 mV using an electrochemical test system (Ivium Technology, Netherlands).

## Conflict of Interest

The authors declare no conflict of interest.

## Supporting information

Supporting Information

Supplemental Video 1

Supplemental Video 2

## Data Availability

The data that support the findings of this study are available in the supplementary material of this article.
