# Supplementary material for: Polymer Electrolyte/Sulfur Double‐Shelled Anisotropic Reduced Graphene Oxide Lamellar Scaffold Enables Stable and High‐Loading Cathode for Quasi‐Solid‐State Lithium‐Sulfur Batteries
Source: Adv Sci (Weinh). 2022 Dec 27;10(6):2205424. doi: 10.1002/advs.202205424 (PMC9951297; doi:10.1002/advs.202205424)
Supplement: Supplementary file 1 — Supporting Information [file ADVS-10-2205424-s003.pdf]

ADVANCED SCIENCE
Open Access

Supporting Information

for *Adv. Sci.*, DOI 10.1002/advs.202205424

Polymer Electrolyte/Sulfur Double-Shelled Anisotropic Reduced Graphene Oxide Lamellar Scaffold Enables Stable and High-Loading Cathode for Quasi-Solid-State Lithium-Sulfur Batteries

Hyun Jung Shin, Sung-Woo Park, Sangbaek Park and Dong-Wan Kim**

Supporting Information

Polymer electrolyte/sulfur double-shelled anisotropic reduced graphene oxide lamellar scaffold enables stable and high-loading cathode for quasi-solid-state lithium sulfur batteries

Hyun Jung Shin, Sung-Woo Park, Sangbaek Park, Dong-Wan Kim**

Supplementary data 1. *Effect of sulfur infiltration methods on the structure and electrochemical properties of VGCF/rGO (VRG)@S cathodes*

Various methods have been used to infiltrate the active sulfur material into VRG foam. First, a suspension in which sulfur and a conductive carbon agent were dispersed in several solvents (ethanol, NMP, and CS_2) was directly dropped onto the VRG foam. As shown in Figure S1a–c, most of the active materials aggregated on the surface and hardly penetrated into the pores of the VRG foam. To achieve efficient infiltration of sulfur into the VRG foam, we examined other approaches with CS_2 solvent, because the lithium sulfur battery (LSB) using the cathode prepared with CS_2 showed a higher initial specific capacity (1000 mAh g^{-1}) in the charge/discharge curve than those using cathodes with other solvents (Figure S1d–f). Although the sulfur/CS_2 solution penetrated well into the VRG foam without conductive carbon agents using the simple drop method (Figure. S2a), the initial specific capacity was very poor (300 mAh g^{-1}) at a current density of 0.1 C (Figure S2d). This result indicates that conductive carbon agents should be included in the suspension to address the poor electrical conductivity of elemental sulfur. To ensure penetration of the sulfur-carbon suspension (S-C/CS_2) into the foam, a decompression method using a syringe was utilized. As a result, the sulfur and carbon particles penetrated the pores of the VRG foam well (Figure S2b). The initial specific capacity of the LSB also increased to 800 mAh g^{-1} (Figure S2e). This implies that the air trapped in the micropores must be removed. Thus, vacuum filtration was performed to achieve sufficient decompression. By sucking air into the bottom, the S-C/CS_2 suspension fully penetrated the VRG foam (Figure S2c). The specific capacities after the first and second cycles were much higher (1000 and 800 mAh g^{-1}, respectively) than those of the syringe method (Figure S2f). However, a significant amount of sulfur and carbon materials were located at the bottom. Therefore, sonication was performed to ensure uniform distribution of sulfur and carbon within the foam and to eliminate air in the pores (Figure 3a).

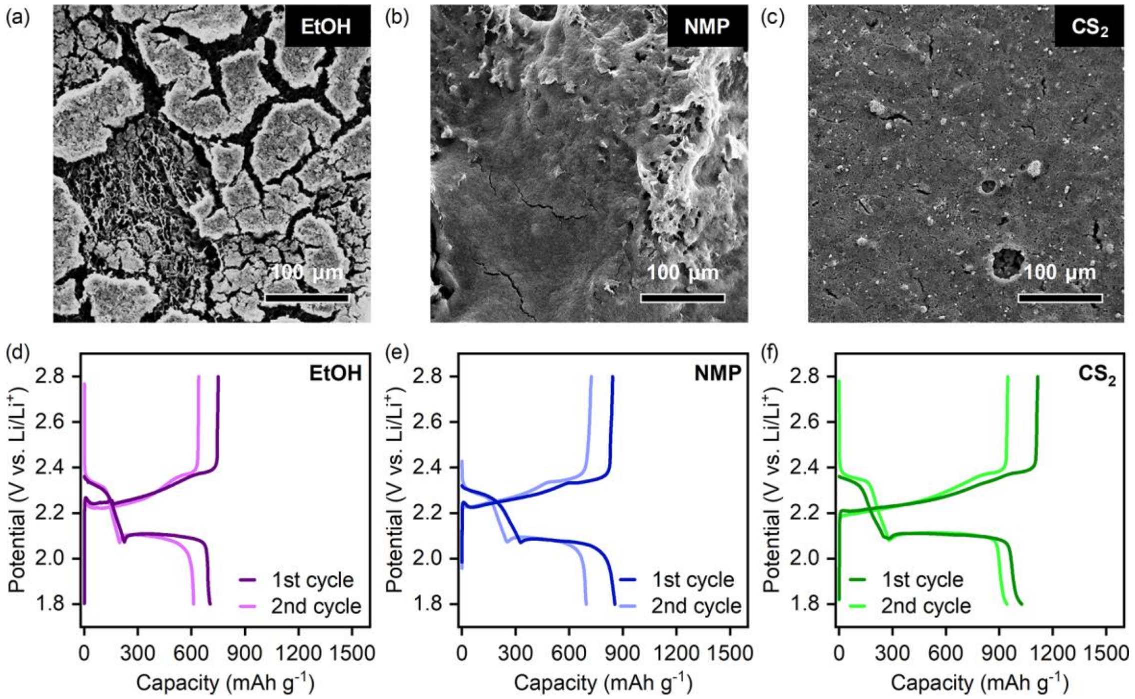

Figure S1. Surface SEM images and GCD curves of LSB cells using VRG@S cathodes fabricated by the simple drop method with various solvents, namely, (a, d) EtOH, (b, e) NMP, and (c, f) CS_2, after the first and second cycles.

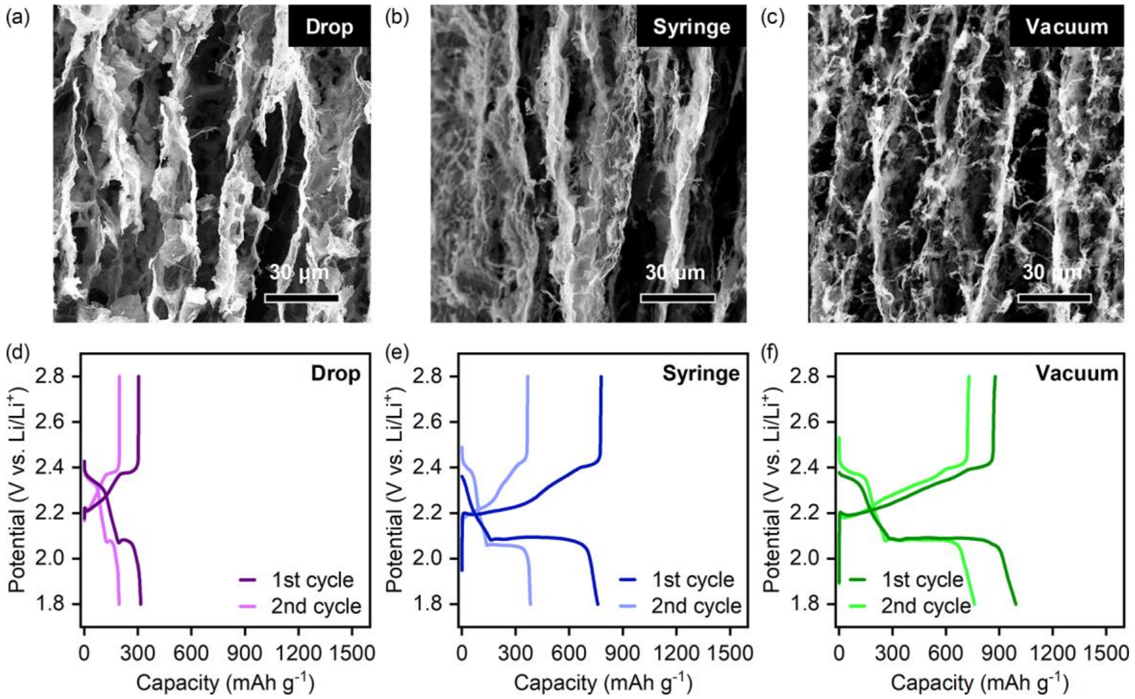

Figure S2. Cross-sectional SEM images and GCD curves of LSB cells using VRG@S cathodes fabricated by various methods, namely, (a, d) simple dropping, (b, e) syringe decompression, and (c, f) vacuum filtration with S-C/CS_2 suspension after the first and second cycles.

Supplementary data 2. *Structural stability test of rGO foam during sonication for the infiltration of sulfur into the anisotropic lamellar scaffold*

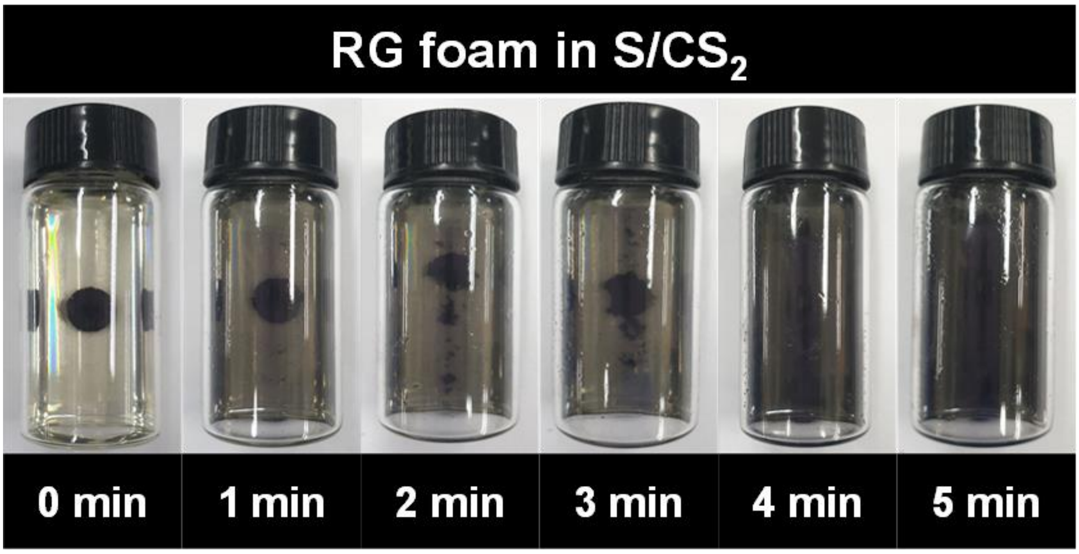

Figure S3. Photographs of the rGO foam in S-C/CS_2 suspension after various sonication durations.

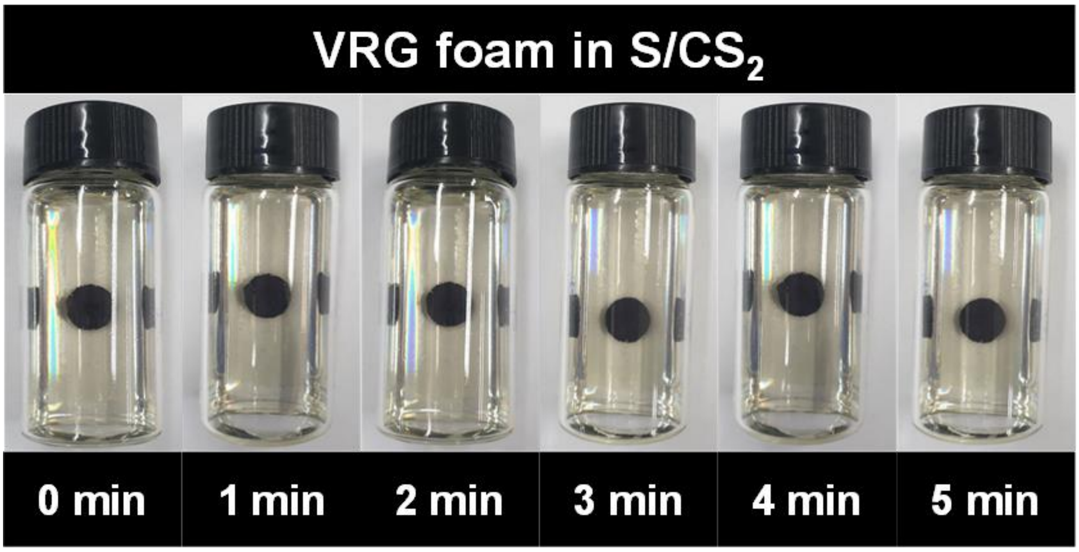

Figure S4. Photographs of the VRG foam in S-C/CS_2 suspension after various sonication durations.

Supplementary data 3. *Coupling type between VGCF and rGO in VRG foam*

In order to verify type of coupling between VGCF and rGO, FT-IR analysis was conducted for the bare rGO, VGCF and VRG (Figure S5). The spectra of three samples were observed between 4000 and 650 cm^{-1}. The related peaks were appeared around 850, 1150, 1400 and 3400 cm^{-1} corresponded to C-C, C-O, C-O-C and O-H stretching, respectively.[42-43] The VGCF exhibited only C-C stretching peak around 1400 cm^{-1}. In contrast, the bare rGO and VRG showed all 4 peaks above-mentioned, and intensity of peaks exhibited that VRG was lower than bare rGO. Generally, when carbon-nanotube and rGO is chemically bonded, O-H stretching around 3400 cm^{-1} reaches down to a lower-wavelength (3000~3100 cm^{-1}), making a shoulder peak.[44] However, such shoulder peak hardly observed in spectra of VRG. It indicates that there is no hydrogen bonding between VGCF and rGO due to the absence of the C-O-H functional group on the surface of VGCF.[45] Therefore, it suggests that the type of coupling between VGCF and rGO is purely mechanical mixing without chemical binding.

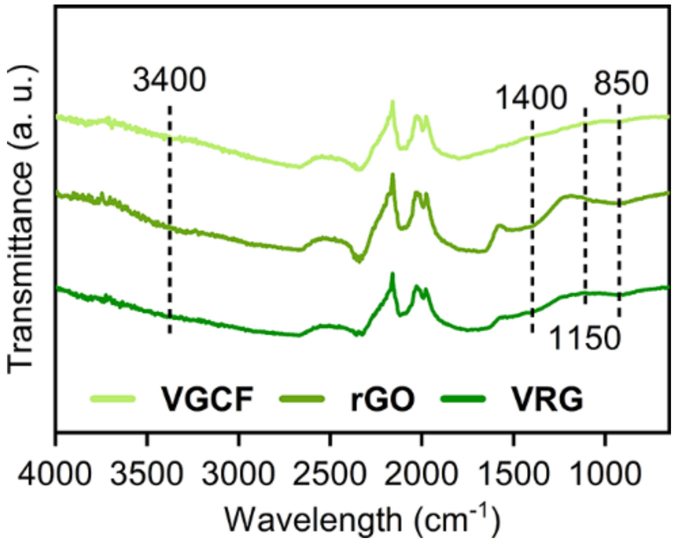

Figure S5. FT-IR spectra of VGCF, bare rGO and VRG.

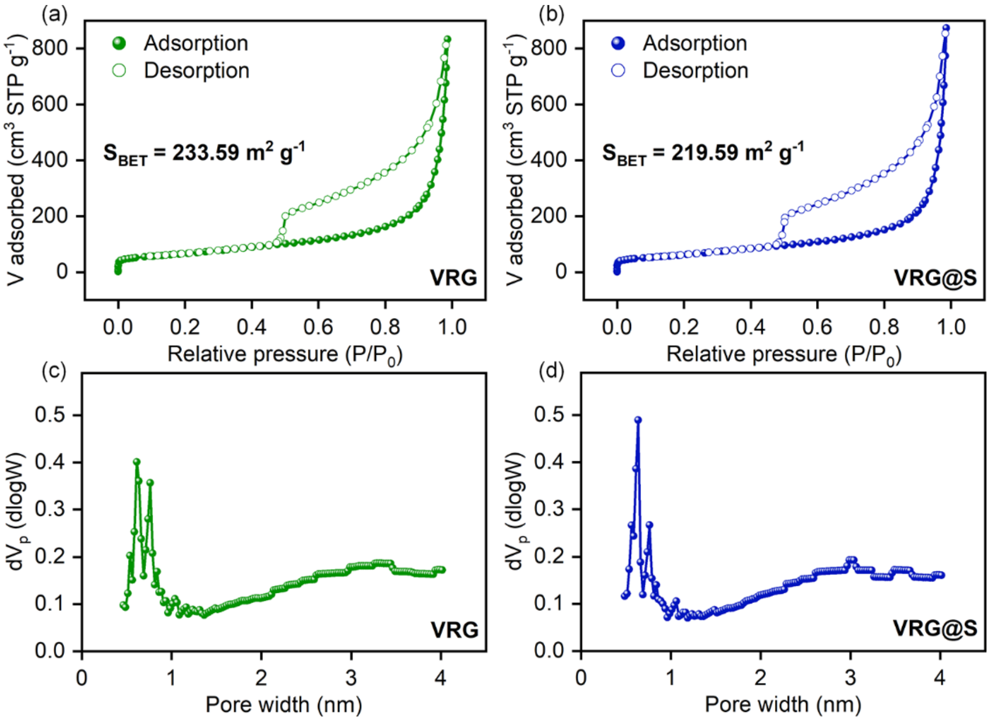

Figure S6. (a-b) N_2 isotherm hysteresis and (c-d) pore distribution results for (a, c) as-prepared VRG foam and (b, d) VRG foam after sulfur infiltration (VRG@S).

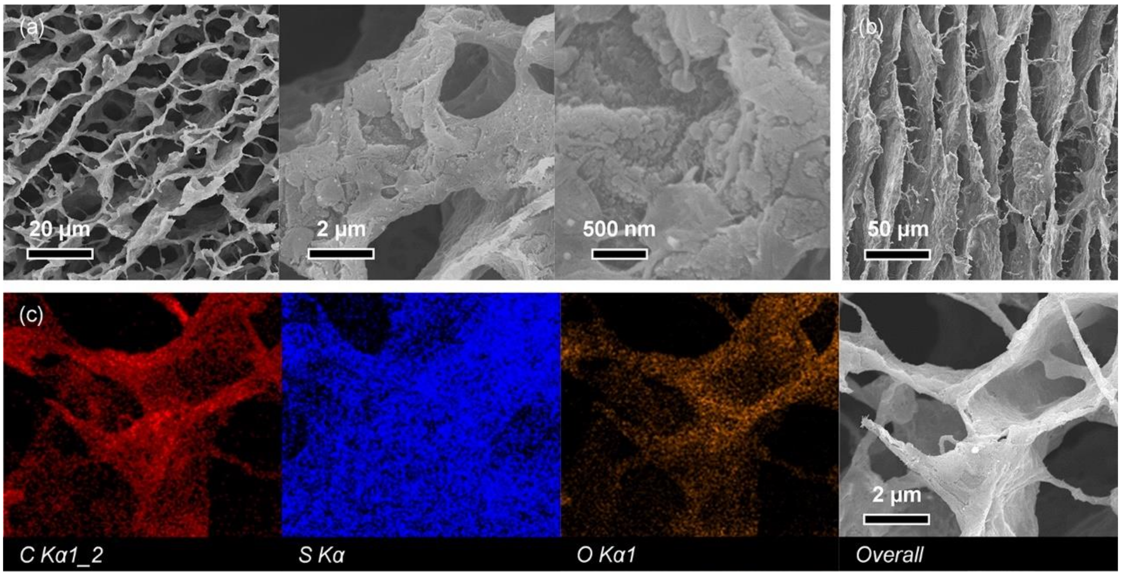

Figure S7. (a) Planar and (b) cross-sectional SEM images of the VRG@S foam after 100 cycles at 1 C. (c) EDS elemental mapping of the VRG@S foam after 100 cycles at 1 C.

Supplementary data 4. *Effect of PEO-based polymer electrolyte (PPE) infiltration methods on the structure and electrochemical properties of VRG@S@PPE cathodes*

PPE was coated onto the surface of the VRG@S foam. Inspired by the success of sulfur infiltration, various methods were evaluated to find the best way to infiltrate PPE into the VRG@S foam. Direct melting of the PPE film can penetrate a large amount of PPE into the foam without void spaces. However, the harsh environment, i.e., high temperature and vacuum conditions, required for the direct melting of PPE induced the sublimation of sulfur in the VRG@S foam. The foam structure also collapsed because of an excess of PPE. Thus, PPE was dissolved in acetonitrile and then coated onto the surface of the VRG@S foam (Figure S8). First, the PPE solution was dropped directly onto the VRG@S foam. As a result, PPE hardly penetrated into the foam and mostly accumulated on the surface, blocking almost all the pores (Figure S8a). Accordingly, the capacity was as low as approximately 300 mAh g^{-1} at a rate of 0.1 C (Figure S8d), because sulfur utilization was reduced by the thick PPE layer deposited on the electrode surface. As with the previous sulfur infiltration approach, decompression was required to penetrate PPE into the foam without accumulation on the surface. Thus, decompression using a syringe was carried out for PPE infiltration. The PPE solution was forcibly penetrated into the foam while removing air in the pores through decompression using a syringe. Although this method better than the simple drop method, a large amount of PPE still barely infiltrated the foam and was accumulated on the surface (Figure S8b). Although the discharge capacity of the corresponding cathode was relatively at approximately 1000 mAh g^{-1} after the first cycle, the charging process was insecure, and subsequently, overcharging occurred during the second cycle (Figure S8e). Therefore, to infiltrate the PPE more effectively, the VRG@S foam was soaked in PPE solution and exposed to a vacuum environment. Consequently, the amount of PPE accumulated on the surface was significantly reduced, and the porous surface of the foam was maintained (Figure S8c). The resulting cathode showed a considerably high capacity (1600 mAh g^{-1}) during the first cycle of the LSB (Figure S8f), although poor capacity retention was observed owing to the thick PPE coating. The capacity retention was improved by reducing the PPE coating amount from 30 to 10 mg (Figure S9). The capacity of 10 mg PPE-coated VRG@S only reduced from 1200 to 1100 mAh g^{-1} during the first three cycles and remained at that value even after the next 10 cycles, which indicates that the initial capacity retention was better than that of bare VRG@S foam without PPE.

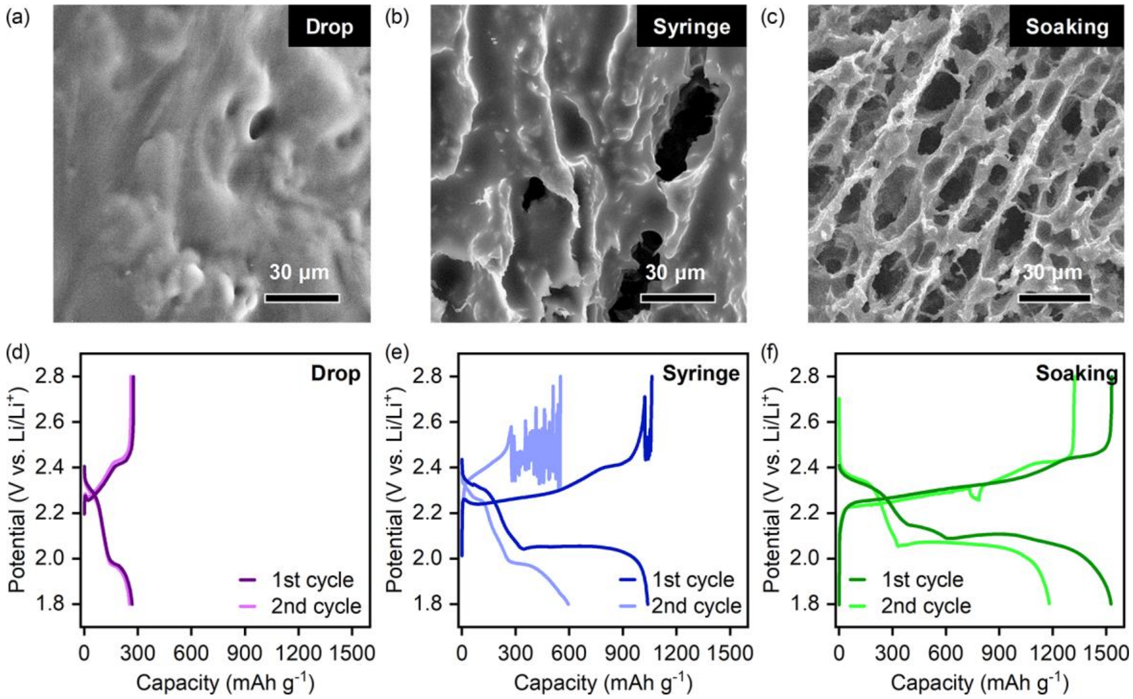

Figure S8. Surface SEM images and GCD curves of LSB cells using VRG@S@PPE cathodes fabricated by various methods, namely, (a, d) simple dropping, (b, e) syringe decompression, and (c, f) soaking and vacuum filtration using PPE solution after the first and second cycles.

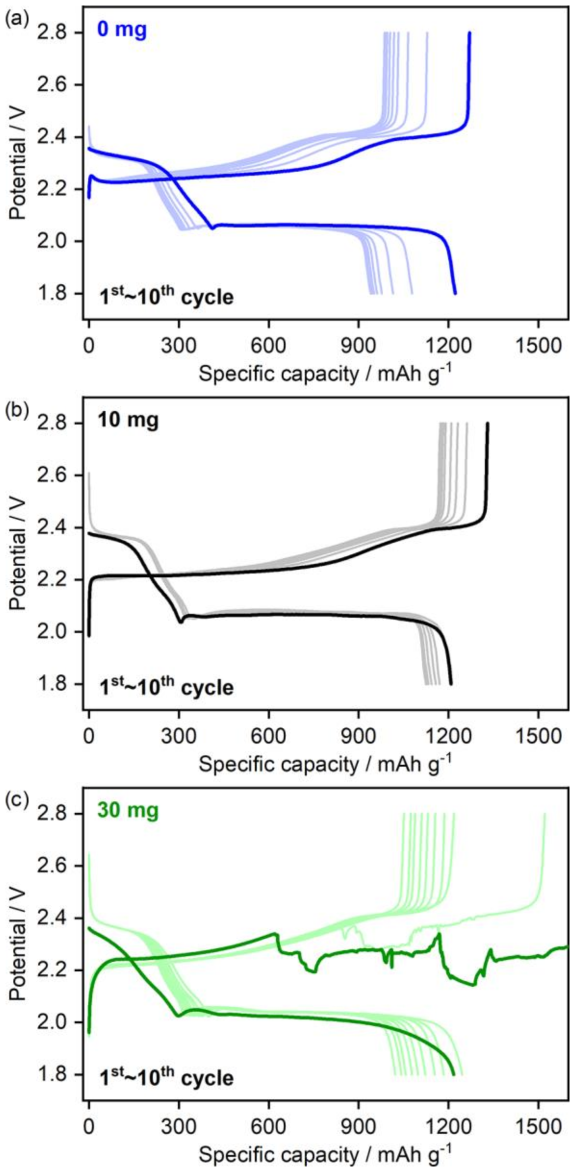

Figure S9. GCD curves of QSSLSBs with VRG@S@PPE cathodes fabricated via soaking and vacuum filtration with (a) 0, (b) 10, and (c) 30 mg of PPE coating for the 1st–10th cycles.

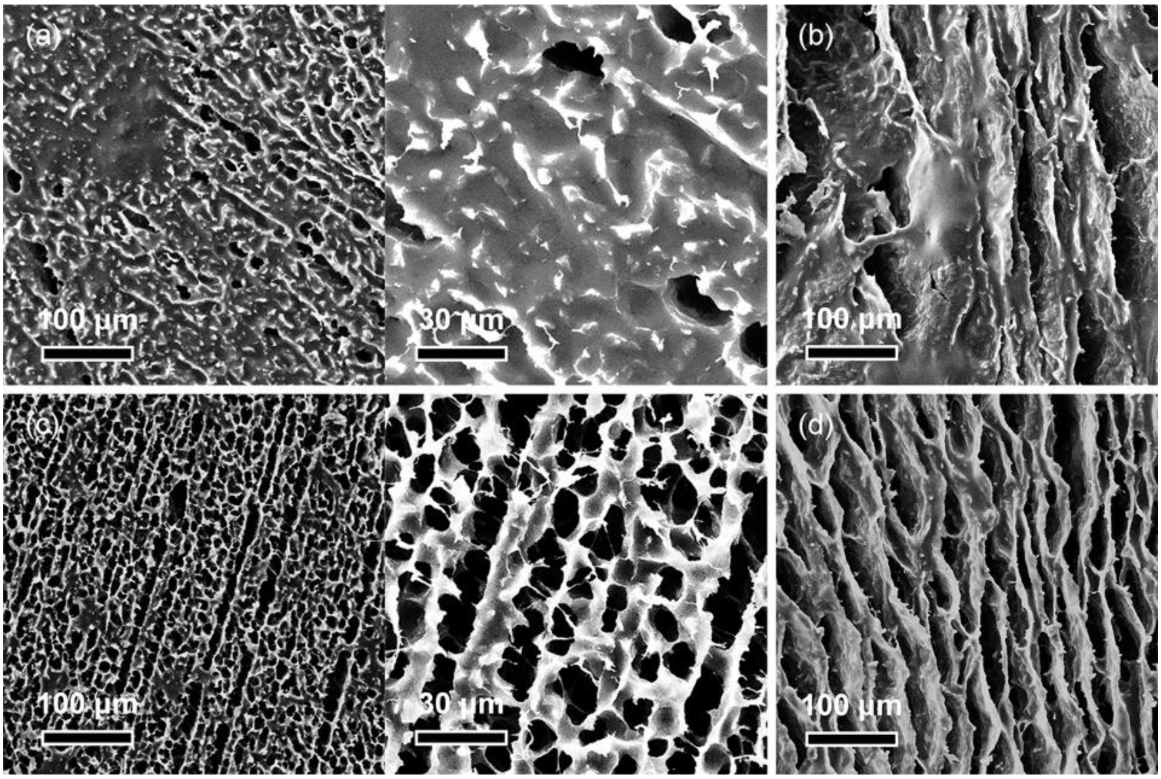

Figure S10. Low-magnification (a,c) planar and (b,d) cross-section SEM image of the (a-b) VRG@PPE by soaking/vacuum filtration method and (c-d) VRG@PPE by sonication method. Here, the infiltrated PPE mass were fixed to 30 mg, to confirm the advantage of sonication method more clearly. When the soaking/vacuum filtration method was used, a large amount of PPE that had not penetrated was accumulated on the surface and blocked the pores. In addition, PPE was non-uniformly aggregated inside the structure. In contrast, not only PPE was hardly stacked but also pores were clearly maintained without blocking in surface of VRG via the sonication method, where PPE which infiltrated internal structure was also uniformly coated on rGO sheet without agglomeration.

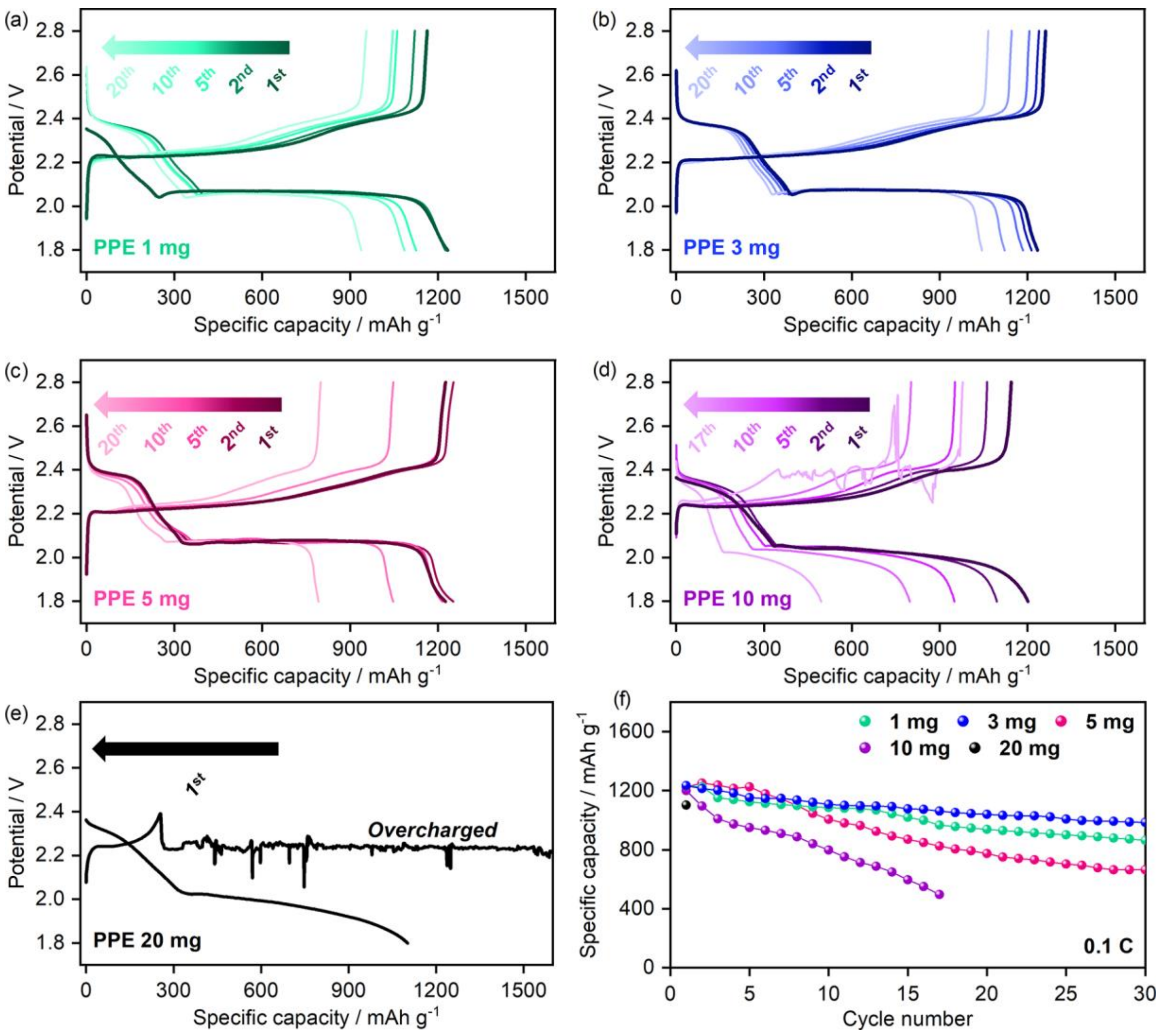

Figure S11. GCD curves of QSSLSBs with VRG@S@PPE cathodes fabricated via sonication infiltration with (a) 1, (b) 3, (c) 5, (d) 10, and (e) 20 mg of PPE coating for the 1st–20th cycles; (f) corresponding cycling plots at 0.1 C.

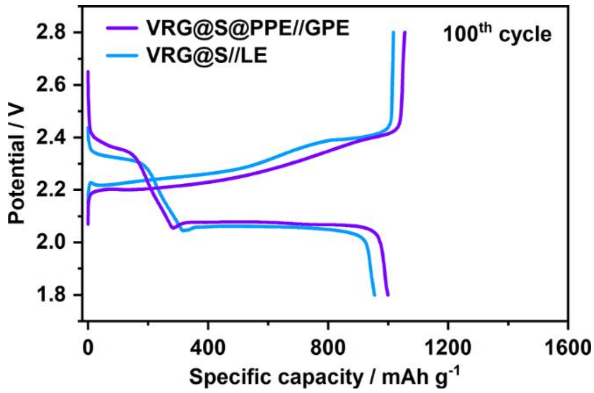

Figure S12. The GCD curves of the 100th cycles of VRG@S@PPE//GPE and VRG@S//LE at 0.1 C.

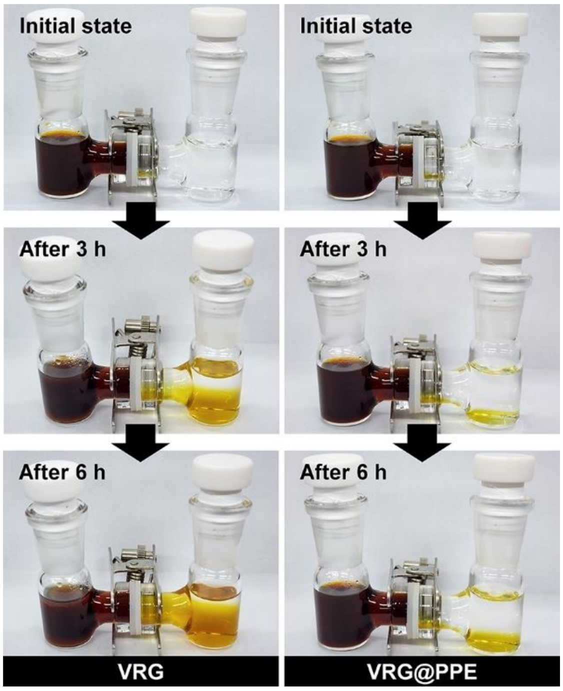

Figure S13. Time-dependent LPS diffusion test of VRG and VRG@PPE using H-type glass cell. LPS diffusion through VRG and VRG@PPE in H-type glass cells were observed at fresh-state, after 3 h and after 6 h.

Table S1. Electrochemical performances of previously reported carbon-based 3D-structured high-loading sulfur electrodes for LSBs

Material	Sulfur loading (mg cm^{-2})	C-rate	Cycle number	Discharge capacity (mAh g^{-1})	Reference
VGCF@rGO@S	6	0.1 C<br>1 C	1st / 100th	1222 / 998<br>854 / 825	This work
Carbon nanofiber	4.8	0.5 C	1st / 100th	400 / 600	[46]
MWCNT	1.25	0.1 C	1st / 100th	948 / 635	[47]
Micron-porous graphene foam	7	0.1 C	1st / 50th	878 / 513	[48]
Graphene foam nested aerogel	9.8	0.2 C	1st / 350th	1000 / 645	[23]
rGO-S composite film	1.5	0.1 C	1st / 300th	1050 / 668	[49]
CNF-S	6	0.1 C	1st / 100th	1036 / 812	[50]
S@CNTs/Co-NC	4.65	0.1 C	1st / 50th	1115 / 811	[51]
Mxene/rGO	6	0.1 C	30th	879	[52]
Porous carbon nanofiber	5.6	0.1 C	1st / 150th	1100 / 432	[53]
N-doped porous carbon foam/S	6	0.2 C	1st / 100th	780 / 621	[54]
Multi-layered sulfur-carbon nanotube film	3.85	0.2 C	1st / 100th	837 / 643	[55]
Reduced graphene oxide-sulfur composite	5.8	0.2 C	1st / 100th	1131 / 792	[56]
Graphene/carbon nanotubes-sulfur	2.46	0.5 C	1st / 100th	1343 / 900	[57]
Semi-graphitic ordered mesoporous carbon with metal/nitrogen doping	5.98	1 C	1st / 120th	923 / 715	[58]

Table S2. The simulation parameters of the EIS results for rGO-S powder and VRG@S foam measured at the first discharged state at room temperature, and for VRG@S foam and VRG@S@PPE measured at the first discharged state and the discharged state after 100 cycles at 50 °C.

Cell	Rs (Ohm)	R_{CT} (Ohm)
rGO-S powder (1st discharge, room temperature)	8.17	44.80
VRG@S (1st discharge, room temperature)	4.46	4.14
VRG@S // LE (1st discharge, 50 °C)	4.50	3.29
VRG@S // LE (after 100th cycles, 50 °C)	6.20	20.54
VRG@S@PPE // GPE (1st discharge, 50 °C)	4.57	5.17
VRG@S@PPE // GPE (after 100th cycles, 50 °C)	6.22	17.53

Table S3. Electrochemical performances of previously reported PEO-introduced polymer-electrolyte-based LSBs

Polymer electrolyte	Electrolyte type	Temperature (ºC)	C-rate	Cycle number	Discharge capacity (mAh g^{-1})	Reference
PEO/LiTFSI coated Celgard	GPE	50	0.1 C	1st / 100th	1207 / 1005	This work
PEO-$Li_{10}SnP_2S_{12}$	SPE	50	0.1 C	1st / 50th	330 / 800	[59]
PEO/LLTO-CNF/S	SPE	25	0.05 C	1st / 50th	380 / 415	[60]
PEO/LiTFSI coated commercial separator	GPE	25	0.1 C	1st / 100th	1182 / 648	[34]
PTFE@LLZO@PEO	SPE	30	0.1 C	1st / 100th	655 / 568	[61]
PEO/LiTFSI coated PP membrane	GPE	25	0.1 C	1st / 100th	1212 / 534	[31]
PEO-S coated Celgard	GPE	25	-	1st / 180th	1200 / 700	[10]
PAN-PEO-LATP	GPE	25	0.1 C	1st / 100th	903 / 714	[2]

Table S4. List of abbreviation used in the paper.

Abbreviation	Definition
LIBs	Lithium-ion batteries
LSBs	Lithium-sulfur batteries
SPE	Solid polymer electrolyte
GPE	Gel polymer electrolyte
QSSLSBs	Quasi-solid-state lithium-sulfur batteries
PPE	PEO-based polymer electrolyte
PEO	Poly(ethylene oxide)
PVDF	Poly(vinylidene fluoride)
LiTFSI	Lithium bis(trifluoromethanesulfonyl)
VGCF	Vapor grown carbon fiber
rGO	Reduced graphene oxide
VG	VGCF/GO
VRG	VGCF/rGO
VRG@S	VGCF/rGO/sulfur
VRG@S_bMD	VRG@S before melting diffusion
VRG@S@PPE	PPE infiltrated VRG@S
VRG@S@PPE//GPE	VRG@S@PPE with PPE film separator
VRG@S//LE	VRG@S with liquid electrolyte

References

[42] M. T. Caccamo, G. Mavilia, S. Magazù, Appl. Sci. 2020, 10, 8159.

[43] L.Wang, N. Liu, Z. Guo, D. Wu,W. Chen, Z. Chang, Q. Yuan,M.Hui, J. Wang, Materials 2016, 9, 206.

[44] J. D. Nunez, A. M. Benito, S. Rouziere, P. Launois, R. Arenal, P. M. Ajayan, W. K. Maser, Chem. Sci. 2017, 8, 4987.

[45] G.Wei, K. Shirai,H. Saitoh, T. Yamauchi, N. Tsubokawa, Carbon 2004, 42, 1923.

[46] X. Zhao, M. Kim, Y. Liu, H.-J. Ahn, K.-W. Kim, K.-K. Cho, J.-H. Ahn, Carbon 2018, 128, 138.

[47] A. B. Kanagaraj, P. Chaturvedi, Y. Kim, D. S. Choi, Mater. Lett. 2022, 306, 130900.

[48] L. Lu, J. T. M. De Hosson, Y. Pei, Carbon 2019, 144, 713.

[49] S. Luo, M. Yao, S. Lei, P. Yan, X.Wei, X.Wang, L. Liu, Z. Niu, Nanoscale 2017, 9, 4646.

[50] J. H. Yun, J. H. Kim, D. K. Kim, H. W. Lee, Nano Lett. 2018, 18, 475.

[51] S. Luo, W. Sun, J. Ke, Y. Wang, S. Liu, X. Hong, Y. Li, Y. Chen, W. Xie, C. Zheng, Nanoscale 2018, 10, 22601.

[52] J. Song, X. Guo, J. Zhang, Y. Chen, C. Zhang, L. Luo, F.Wang, G.Wang, J. Mater. Chem. A 2019, 7, 6507.

[53] R. Li, Y. Dai, W. Zhu, M. Xiao, Z. Dong, Z. Yu,H. Xiao, T. Yang, Ionics 2022, 28, 2155.

[54] Q. Zhu, H. Deng, Q. Su, G. Du, Y. Yu, S. Ma, B. Xu, Electrochim. Acta 2019, 293, 19.

[55] W. Y. Lee, E. M. Jin, J. S. Cho, D.-W. Kang, B. Jin, S. M. Jeong, Energy 2020, 212, 118779.

[56] Y. Liu, M. Yao, L. Zhang, Z. Niu, J. Energy Chem. 2019, 38, 199.

[57] H. Shi, X. Zhao, Z.-S. Wu, Y. Dong, P. Lu, J. Chen, W. Ren, H.-M. Cheng, X. Bao, Nano Energy 2019, 60, 743.

[58] H. Li, D. Liu, X. Zhu, D. Qu, Z. Xie, J. Li, H. Tang, D. Zheng, D. Qu, Nano Energy 2020, 73, 104763.

[59] X. Li, D. Wang, H. Wang, H. Yan, Z. Gong, Y. Yang, ACS Appl. Mater. Interfaces 2019, 11, 22745.

[60] P. Zhu, C. Yan, J. Zhu, J. Zang, Y. Li, H. Jia, X. Dong, Z. Du, C. Zhang, N. Wu, M. Dirican, X. Zhang, Energy Storage Mater. 2019, 17, 220.

[61] Z.-C. Li, T.-Y. Li, Y.-R. Deng,W.-H. Tang, X.-D.Wang, J.-L. Yang, Q. Liu, L. Zhang, Q. Wang, R.-P. Liu, Rare Met. 2022, 41, 2834.
